# Allograft augmentation in proximal humerus fractures

**DOI:** 10.1007/s00064-016-0446-8

**Published:** 2016-05-24

**Authors:** S. A. Euler, F. S. Kralinger, C. Hengg, M. Wambacher, M. Blauth

**Affiliations:** Klinik für Unfallchirurgie und Sporttraumatologie, Medizinische Universität Innsbruck, Anichstr. 35, 6020 Innsbruck, Austria; Unfallchirurgische Abteilung, Wilhelminenspital Wien, Montlearstr. 37, Vienna, 1160 Austria

**Keywords:** Proximal humerus fracture, Displacement, Plate fixation, Allograft, Patient non-compliance, Proximale Humerusfraktur, Dislokation, Plattenfixierung, Allograft, Patienten-Non-Compliance

## Abstract

**Objective:**

Achieve stable fixation to initially start full range of motion (ROM) and to prevent secondary displacement in unstable fracture patterns and/or weak and osteoporotic bone.

**Indications:**

(Secondarily) displaced proximal humerus fractures (PHF) with an unstable medial hinge and substantial bony deficiency, weak/osteoporotic bone, pre-existing psychiatric illnesses or patient incompliance to obey instructions.

**Contraindications:**

Open/contaminated fractures, systemic immunodeficiency, prior graft-versus-host reaction.

**Surgical technique:**

Deltopectoral approach. Identification of the rotator cuff. Disimpaction and reduction of the fracture, preparation of the situs. Graft preparation. Allografting. Fracture closure. Plate attachment. Definitive plate fixation. Radiological documentation. Postoperative shoulder fixation (sling).

**Postoperative management:**

Cryotherapy, anti-inflammatory medication on demand. Shoulder sling for comfort. Full active physical therapy as tolerated without pain. Postoperative radiographs (anteroposterior, outlet, and axial [as tolerated] views) and clinical follow-up after 6 weeks and 3, 6, and 12 months.

**Results:**

Bony union and allograft incorporation in 9 of 10 noncompliant, high-risk patients (median age 63 years) after a mean follow-up of 28.5 months. The median Constant–Murley Score was 72.0 (range 45–86). Compared to the uninjured contralateral side, flexion was impaired by 13 %, abduction by 14 %, and external rotation by 15 %. Mean correction of the initial varus displacement was 38° (51° preoperatively to 13° postoperatively).

## Zusammenfassung

**Operationsziel:**

Erreichen einer stabilen Osteosynthese bei instabilen Frakturen und/oder schwacher/osteoporotischer Knochenstruktur, um von Beginn an eine volle Bewegungsstabilität (ROM) zu erreichen.

**Indikationen:**

(Sekundär) dislozierte proximale Humerusfrakturen (PHF) mit instabiler medialer Abstützung und substanziellem Substanzdefekt. Schwache/osteoporotische Knochenstruktur. Bestehende Psychosen oder Patienten-Non-Compliance, Instruktionen zu befolgen.

**Kontraindikationen:**

Offene/kontaminierte Frakturen. Systemische Immundefekte. Stattgehabte Abstoßungsreaktion.

**Operationstechnik:**

Deltoideopektoraler Zugang. Identifikation der Rotatorenmanschette. Reposition der Fraktur, Präparation von Situs und Allograft. „Allografting“. Verschluss der Fraktur. Anpassung der Platte. Definitive Fixierung der Platte. Radiologische Dokumentation. Postoperative Fixierung der Schulter im Gurt.

**Weiterbehandlung:**

Kryotherapie. Analgetische Medikation und Schultergurt nach Bedarf. Volle aktive Physiotherapie soweit möglich nach Schmerzvorgabe. Postoperative Röntgenaufnahmen (anteroposterior, outlet view und axial [je nach Patiententoleranz]) und klinisches Follow-up nach 6 Wochen sowie nach 3, 6 und 12 Monaten.

**Ergebnisse:**

Knöcherne Heilung und Einheilung des Allografts bei 9 von 10 nicht compliant Hochrisikopatienten (medianes Alter 63 Jahre) bei einem mittleren Nachuntersuchungszeitraum von 28,5 Monaten. Der mediane Constant-Murley-Score war 72,0 (Spanne 45–86). Verglichen zur unverletzten kontralateralen Seite konnte eine Verbesserung der Flexion von 13 %, der Abduktion von 14 % und der Außenrotation von 15 % gefunden werden. Die mittlere Korrektur der initialen Varusfehlstellung war 38° (51° präoperativ vs. 13° postoperativ).

## Introduction

This technique may reinforce and augment internal plate fixation in displaced proximal humeral factures (PHF) with an unstable medial hinge, especially in weak and osteoporotic bone with substantial loss of the structural bony scaffold. Compared to conventional plate fixation methods, it may not only decisively increase bony stability and prevent secondary fracture displacement, but also allow for full initial range of motion (ROM) [1].

## Surgical principles and objective

To augment surgical fixation and to achieve postoperative stability strong enough to initially start full ROM and to prevent secondary displacement in unstable fracture patterns and/or weak and osteoporotic bone.

## Advantages

- Joint preserving method without artificial material
- Increased stability after open reduction and internal plate fixation of PHF
- Anatomic reduction in cases of substantial bone loss using a biological structural void filler
- Strong structural bony congruency
- No additional surgical approach, wound site, or donor morbidity
- Average technical skills demanded
- Initial full weightbearing and ROM
- Potential prevention of secondary postoperative fracture displacement
- Very low infection rates or graft-versus-host reactions [1, 3]
- Solid bone stock for potential secondary prosthetic interventions

## Disadvantages

- Allogenic bony material
- Potential risk of infection, transmission of diseases and graft-versus-host reaction
- Minimal risk of nonunion
- Minimally increased operation time
- Limited accessibility to allografts
- Increased costs if not derived by in-house bony banks

## Indications

- (Secondarily) displaced 2‑part proximal humerus fractures (PHF) with an unstable medial hinge and substantial bony deficiency
- Cases of weak and osteoporotic bony structure
- Increased risk for secondary displacement due to pre-existing psychiatric illnesses or patient incompliance to obey rules [2, 5]

## Contraindications

- Open or contaminated fractures
- Systemic immunodeficiency
- Running systemic chemotherapy
- Prior graft-versus-host reaction

## Patient information

The following risks are possible:

- Contamination/transmission of diseases [3]
- Graft-versus-host reaction, systemic host rejection
- Implant failure (screw perforation, loosening, breakage, or intolerance)
- Nonunion
- Bony dissolution over time
- Disintegration and secondary displacement
- Re-operation
- Infection, thrombosis, embolism, vascular or nerve damage

## Preoperative work up

- Bilateral shoulder CT and 3D reconstruction to distinctively assess the grade of displacement and/or the size of the bony defect
- Pre-order (in-house bank or third party) of an appropriately sized bony allograft (at least one half of a femoral head)
- Femoral heads seem to be rather nonosteoporotic if derived from a replacement surgery of an arthritic hip
- The allograft should be fresh frozen and test negatively for transmittal diseases, contamination, and infection, no antibiotic treatment or preserving processing to the graft prior to implantation
- Thawing of the fresh frozen graft to room temperature at least 1 h prior to surgery
- Shaving of the complete shoulder region, including axilla
- Single shot intravenous antibiotic administration (bone consistently, at least 30 min prior to the skin cut, i. e., aminopenicillin) [4]

## Instruments

- Bone saw to decorticate the allograft
- Luer-like instruments (Rangeur)

## Anesthesia and positioning

**Fig. 1:**
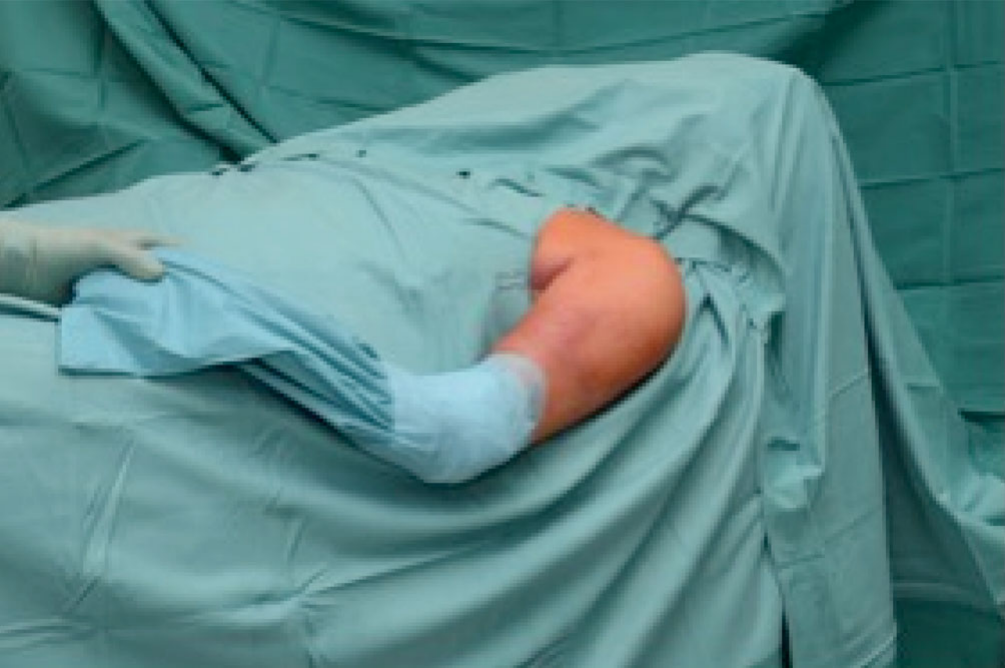
Supine positioning of the patient with the upper body elevated by approximately 20° and the shoulder extending from the table’s edge, allowing the surgeon free manipulation

**Fig. 2:**
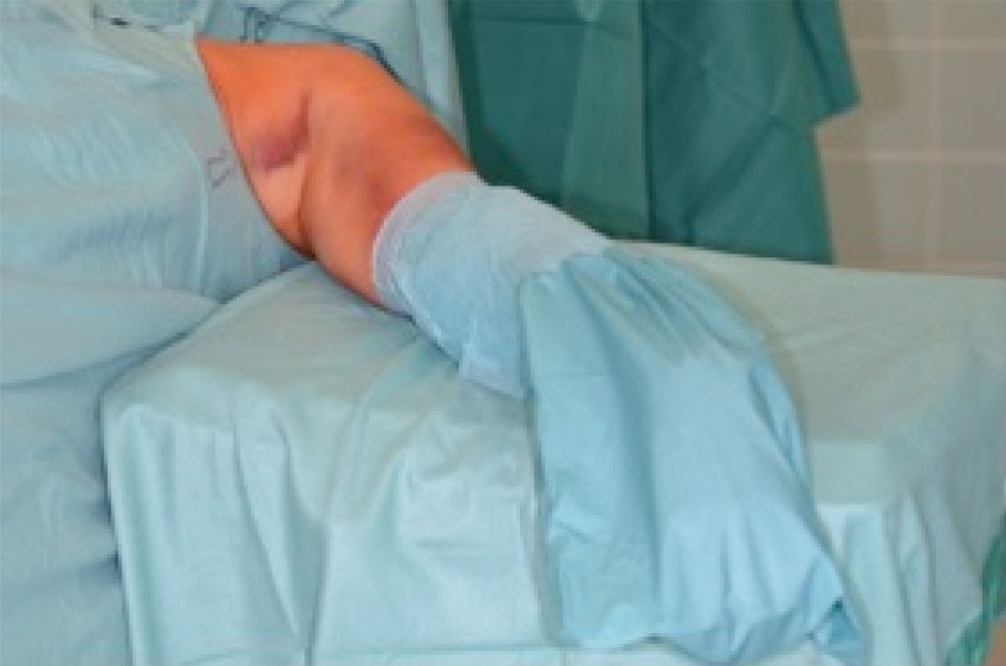
Positioning of the optional mobile arm table (soft surface and edges), adjustable in height and freely movable

- General anesthesia
- Interscalene block (beneficial and recommended, but not mandatory)
- Supine position and mild angulation of the upper body (approximately 20°; Fig. 1)
- Positioning on the edge of the table with the arm freely movable on an optional adjustable table (Fig. 2)
- Regular prepping and wrapping

## Surgical technique

(Figs. 3, 4, 5, 6, 7, 8, 9, 10, 11, 12, 13, 14**,**15**, **16, 17)

**Fig. 3:**
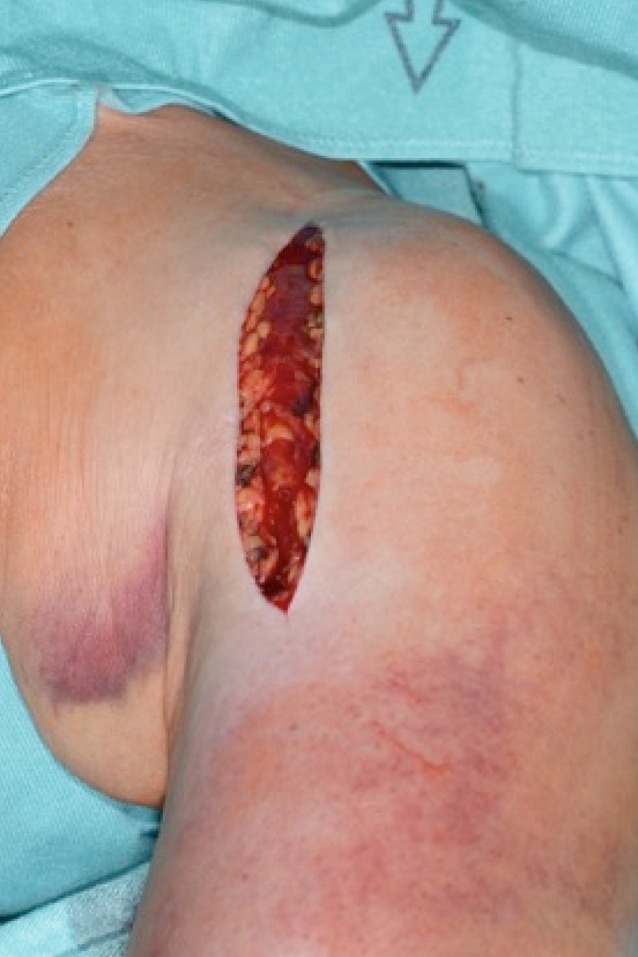
Surgical approach. Deltopectoral approach, 10–12 cm in length. The incision starts distally to the coracoid, continuing distally towards the ventral humerus, orienting just above the medial border of the deltoid muscle, lateral to the axilla. The subcutaneous fatty tissue is then cut to expose the deltopectoral fascia underneath

**Fig. 4:**
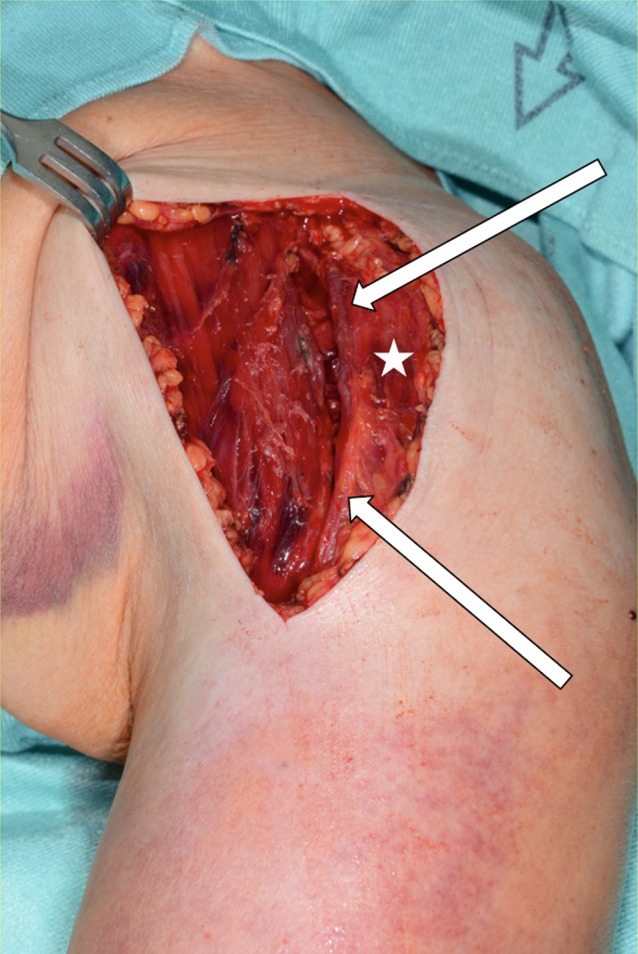
Deep deltopectoral approach. Sharp or blunt skin retractors should be used to display the surgical situs. The cephalic vein is then identified underneath the fascia (*white arrows*), dividing the deltoid muscle laterally, and the pectoralis major muscle medially. Prepping to its medial site, the vein is held laterally, still attached to the deltoid muscle. *White asterisk* indicates the deltoid

**Fig. 5:**
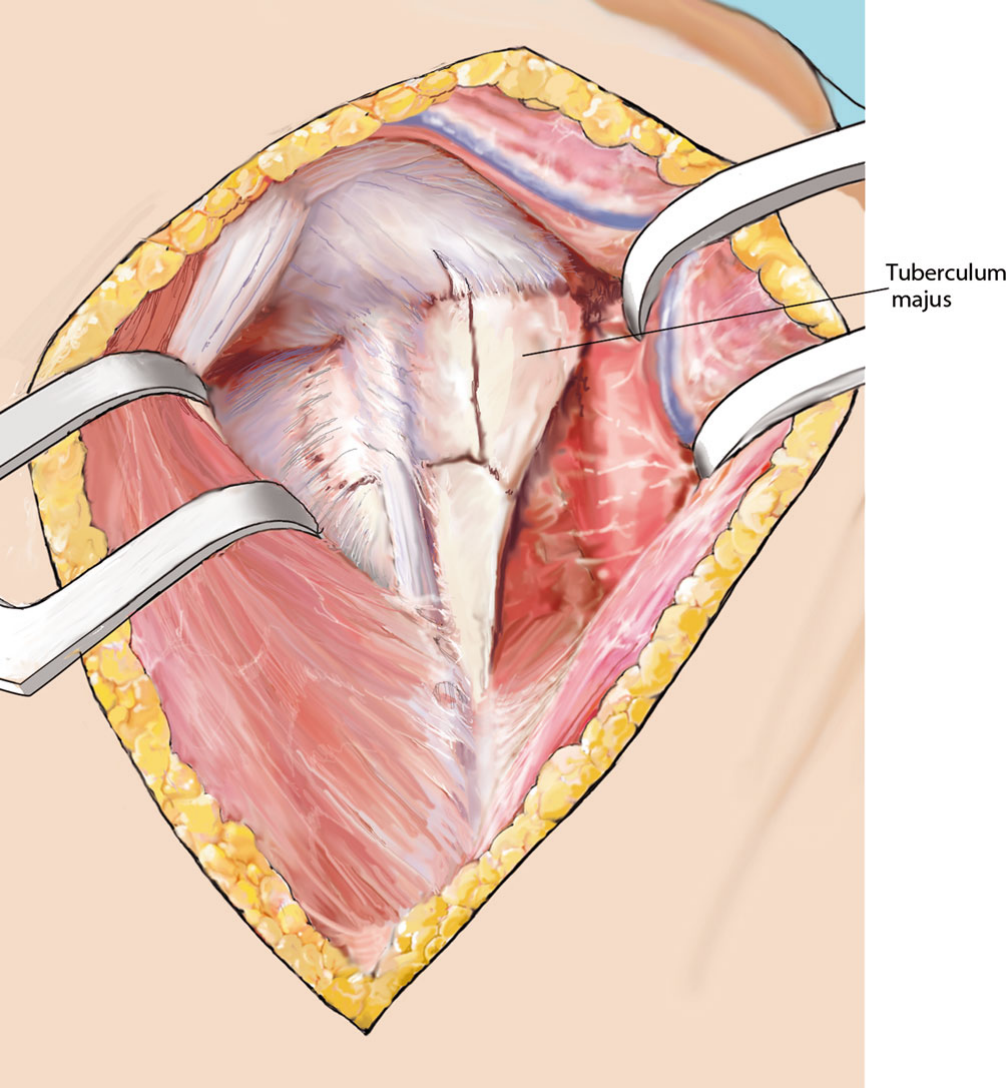
Fracture situs. Identifying the coracoid in the depth, careful blunt division of deltoid and pectoralis major muscles is performed from proximal to distal. Originating from the coracoid, the conjoint tendon may now be identified and retraced medially to display the fracture situs (a four-part fracture of the proximal humerus is shown in the drawing). Special care should be taken not to harm the axillary nerve

**Fig. 6:**
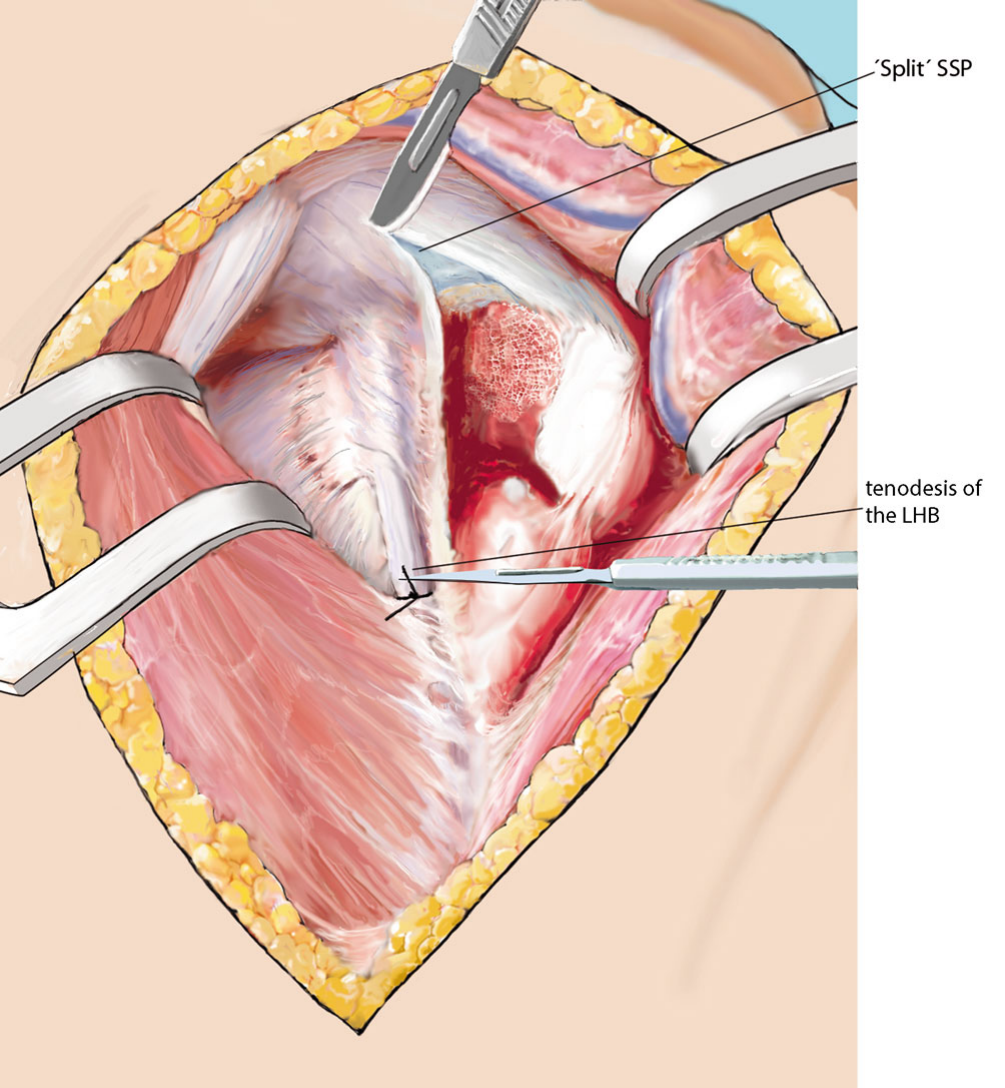
Identification of the rotator cuff. Prior to the reduction of the fracture, the functional components of the rotator cuff have to be identified and looped. Anatomically, the long head of the biceps tendon (LHB) and its bicipital groove on the humerus divides the lesser tuberosity (anterior part of the rotator cuff, subscapularis tendon) from the greater tuberosity (lateroposterior part of the rotator cuff, supraspinatus (SSP) and infraspinatus tendons). Stay sutures at the tendon bone interface are placed to be able to gently manipulate the fragments. Usually, the fracture line between the tuberosities runs about 8–10 mm posterior to the bicipital groove. In cases of four-part fractures, or whenever sutures are crossing the sulcus, the LHB is identified and cut above the pectoralis major’s tendon and sutured to the tendon. Any intertuberosity fracture is followed to the SSP, which is split longitudinally to entry the joint. The proximal portion of the LHB is cut at its origin on the superior glenoid and removed. In case of any three-part fracture (greater tuberosity involved) without a fracture of the bicipital groove, the LHB is not treated at all

**Fig. 7:**
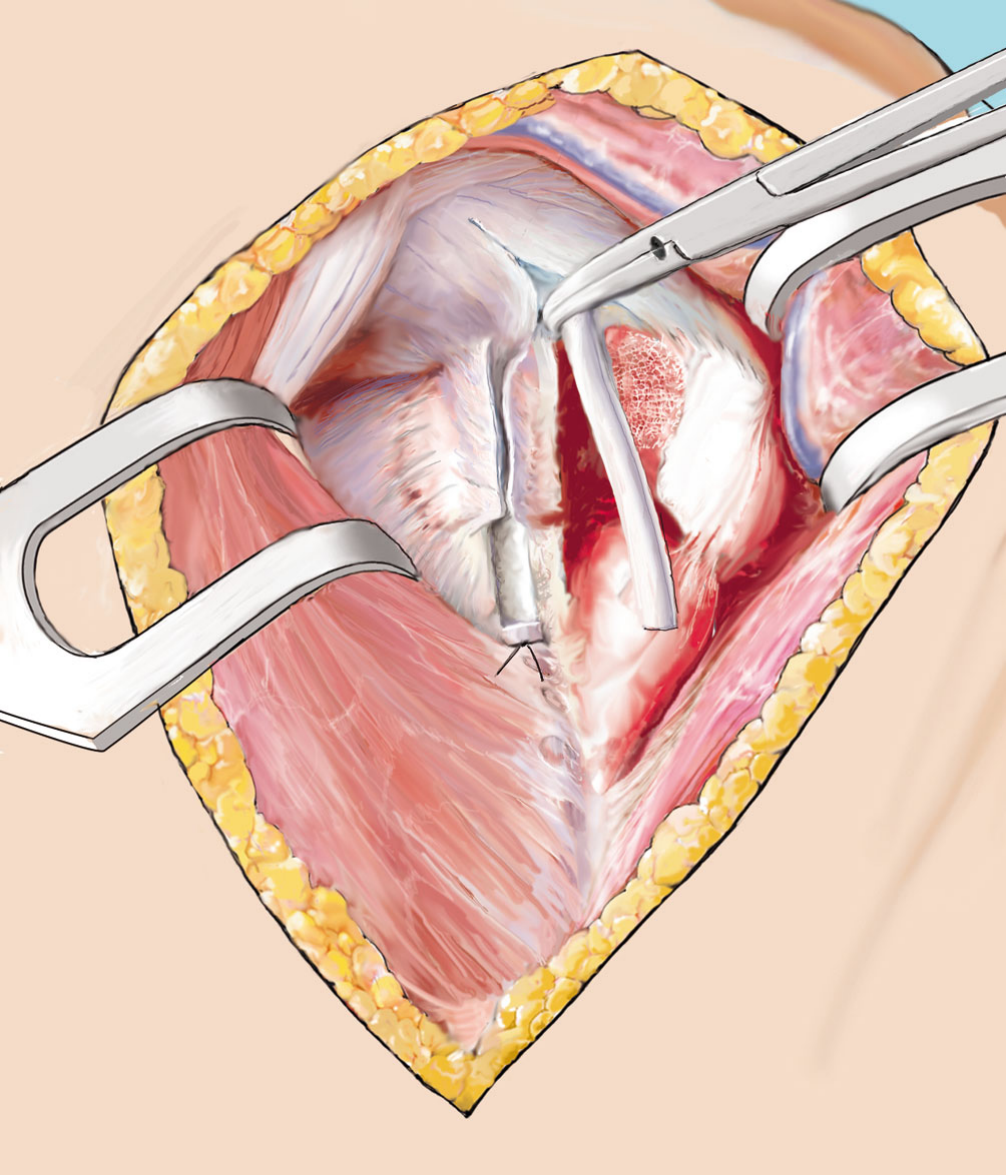
Disimpaction and reduction of the fracture, preparation of the situs. All three functional components of the rotator cuff are looped to later easily close the cuff and secure it to the plate. Fracture displacement and impaction may now be reduced and the fracture is “opened”. Manipulating the elbow, mildly rotating and pulling the shaft component may help to properly reduce the fracture and to align the fracture components. Drawing of the partially “opened” fracture situs. The LHB is followed medially and cut at its origin using a scissors

**Fig. 8:**
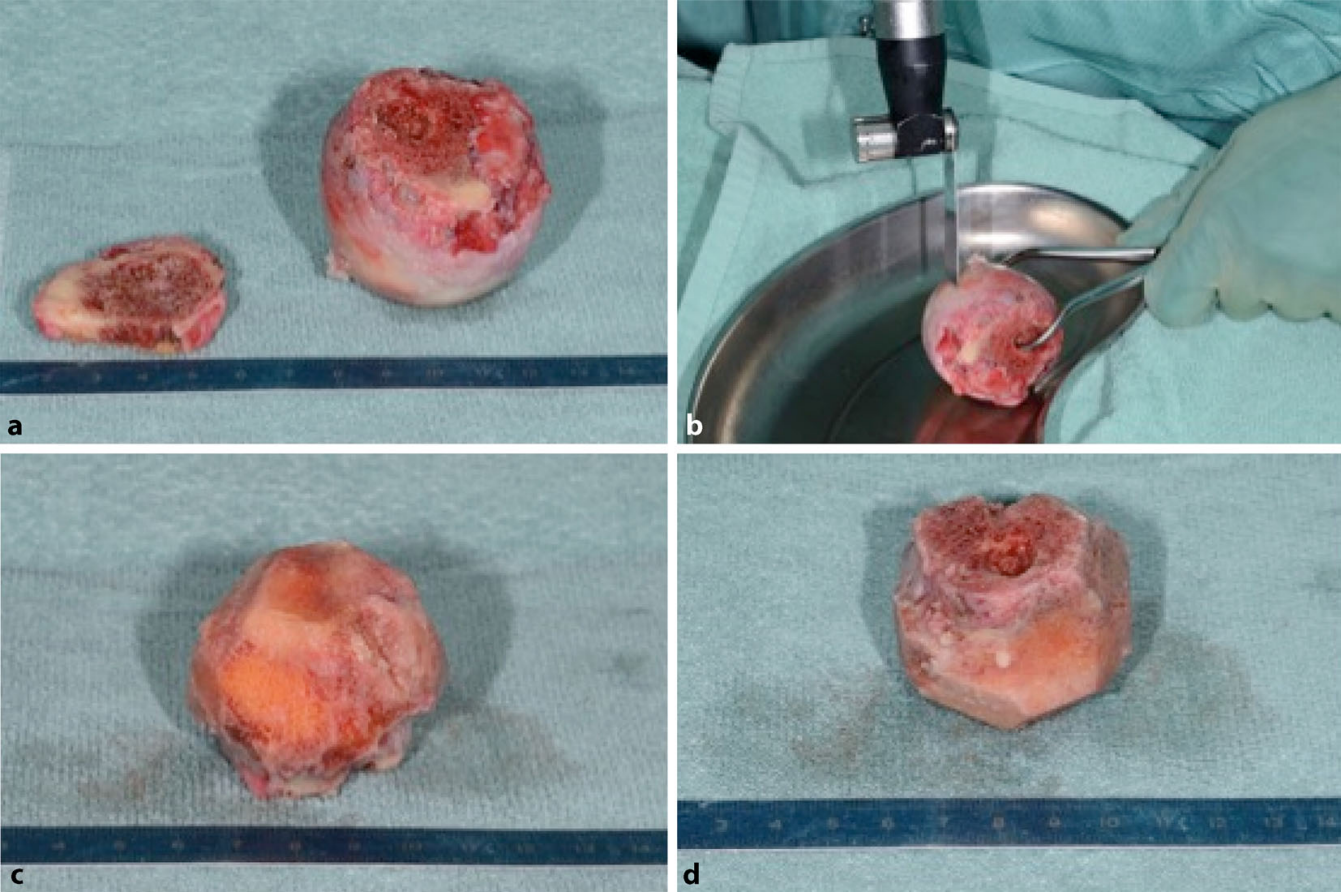
Preparation of the allograft. Any soft tissue or articular cartilage is dissected off the allograft using a saw. To allow for secure positioning within the distal shaft component and to serve as a scaffold with the largest possible surface for the head fragment at the same time, the graft is shaped like a “mushroom” or “Champaign plug”. **a** Fresh frozen partial femoral neck (*left*) and head (*right*) allografts. **b** Dissection of the allograft (off patient). **c,** **d** Pretreated allograft with all cartilage dissected

**Fig. 9:**
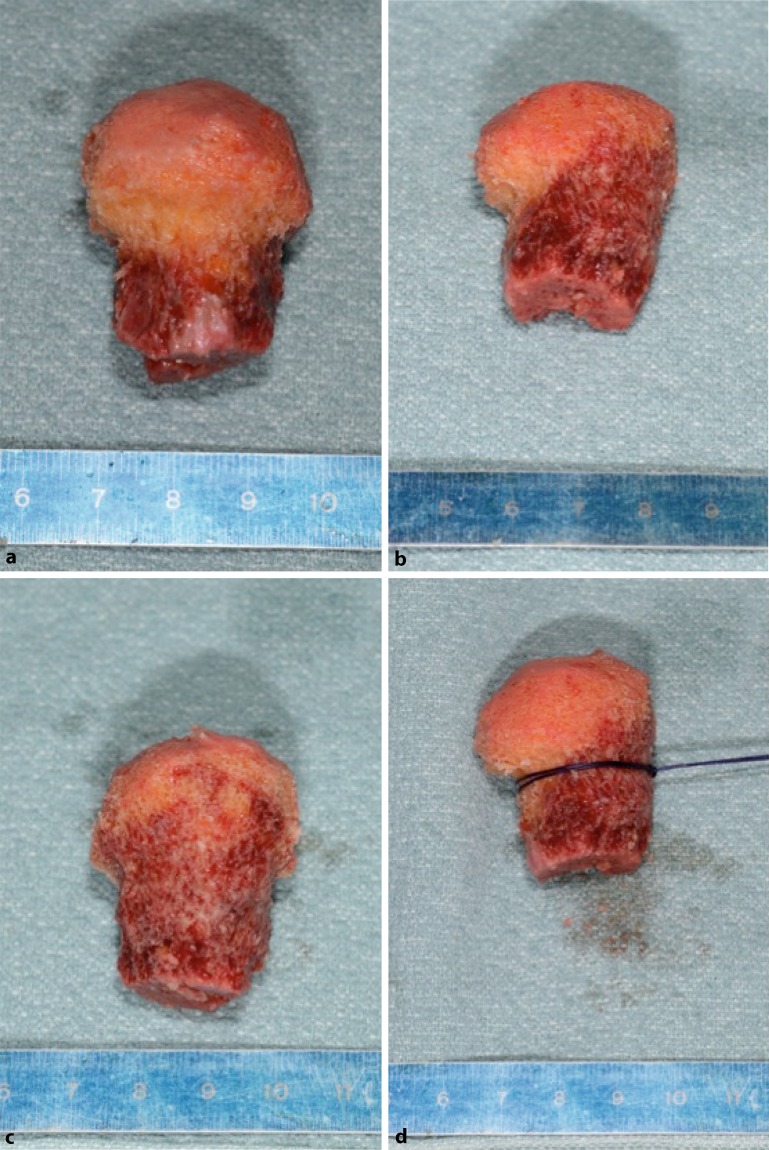
Detailed graft preparation. In the ideal case of a femoral head allograft, the femoral neck may represent the mushroom’s stem, and the femoral head may represent the carrying surface component. Ideally, the dense subchondral zone of the femoral head is preserved to build the strong mushroom’s “hat”. The graft is oversized with approximately 30 mm in width and height. However, individual tailoring of the allograft is necessary. In order to allow easy removal, which will be necessary to exactly shape the graft for the individual defect’s size, No. 1 Vicryl is used to loop and secure the graft around its neck. **a,** **b,** **c,** **d** “Mushroom”-shaped allograft, approximately 30 mm in width and height. **d** Looped and secured using No. 1 Vicryl

**Fig. 10:**
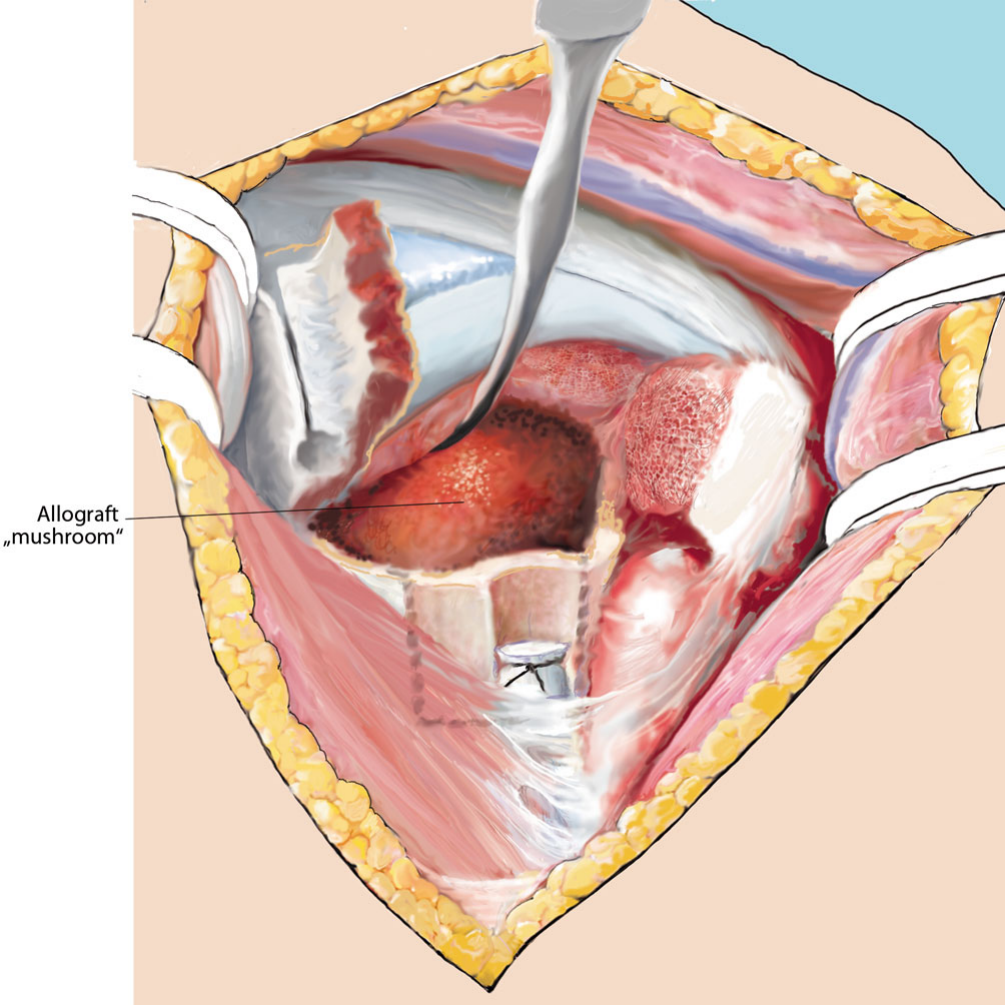
“Allografting”. The graft is then implanted into the fracture situs, with its stem (*dashed line*) facing the humeral shaft, and its roof filling the hollow humeral head. Using Luer forceps, the graft is adjusted. This step is crucial, since the allograft has to be removed several times to perfectly tailor it to the individual fracture and defect site without breaking it

**Fig. 11:**
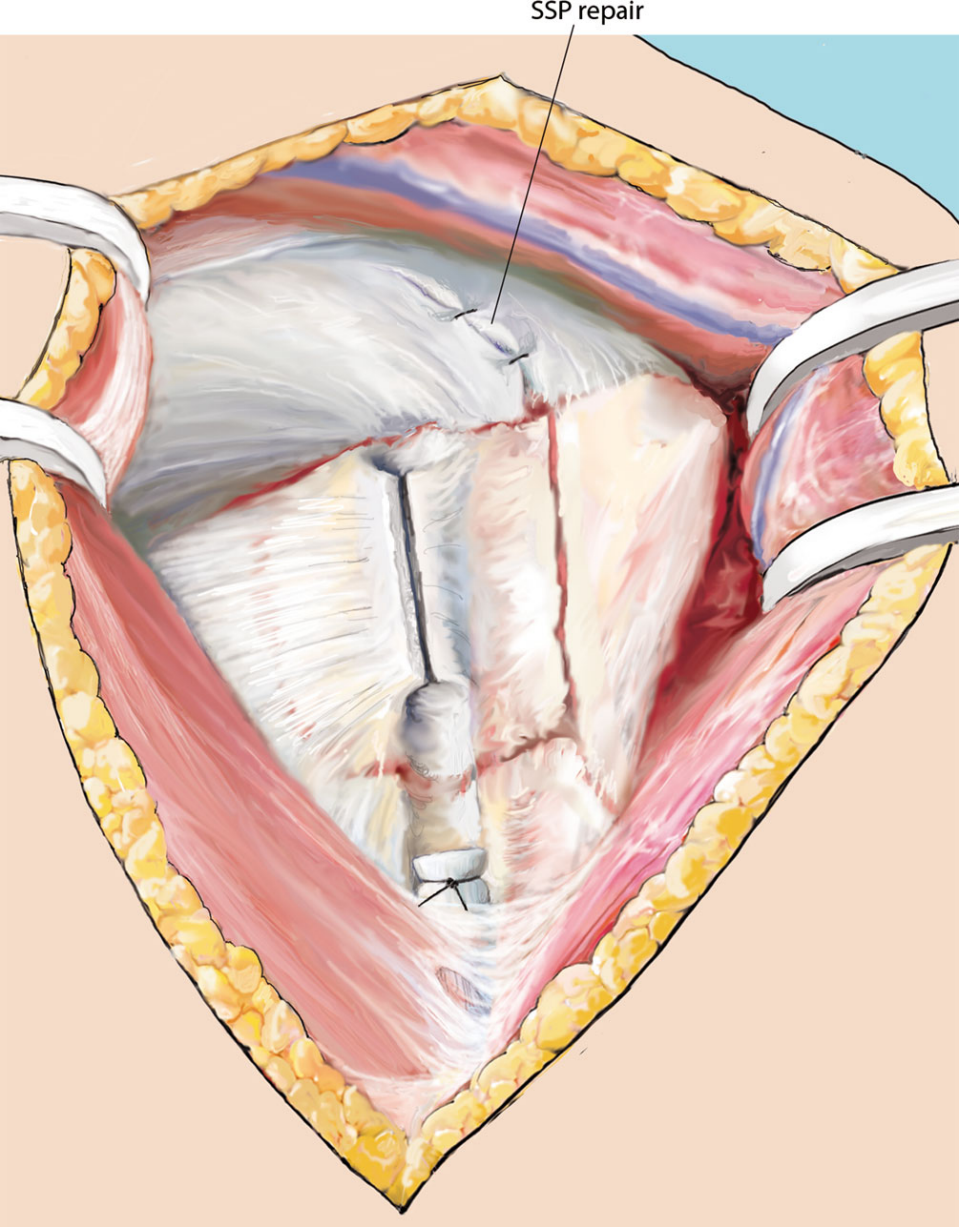
Fracture “closure”. Once the allograft fits macroscopically, the head and the tuberosities are reduced using No. 1 Vicryl wires. A 2.0 mm Kirschner wire is used to temporarily retain the reduction. SSP split is repaired

**Fig. 12:**
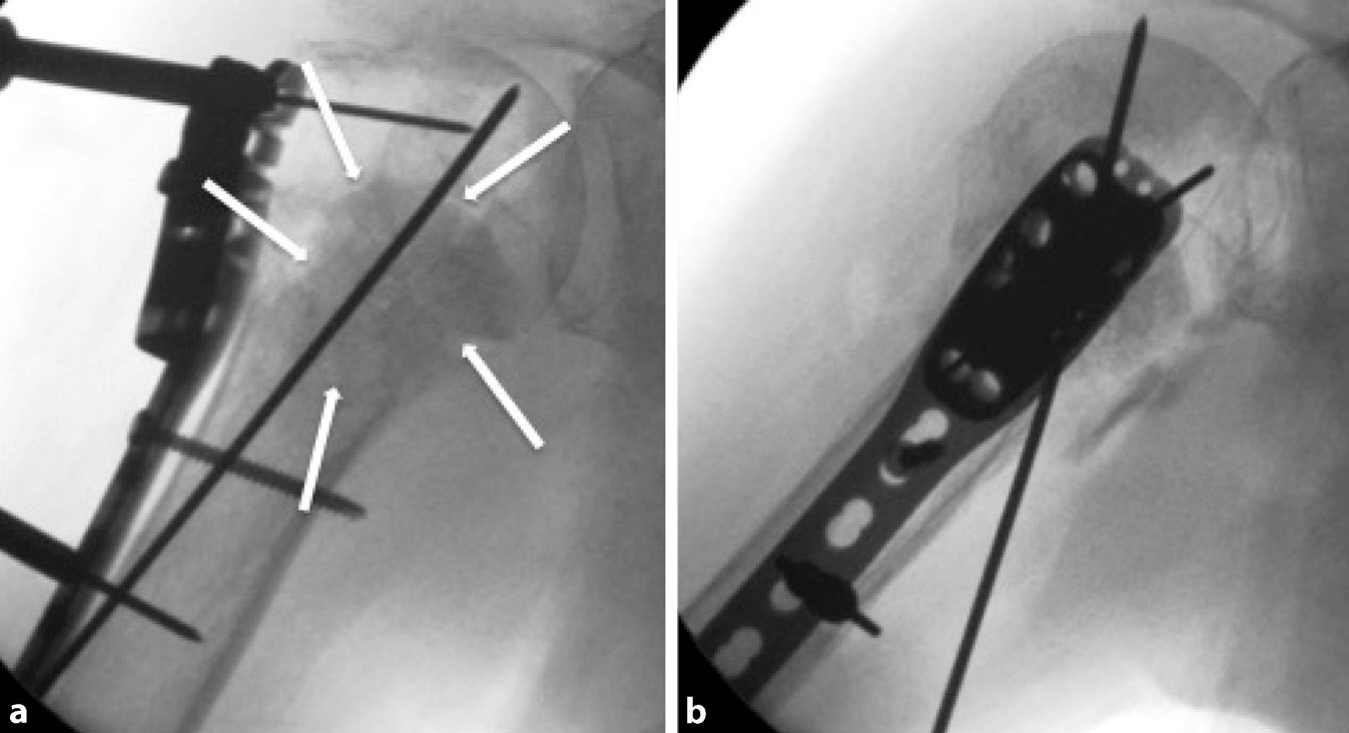
Intraoperative fluoroscopy. Anteroposterior (**a**) and axial (**b**) fluoroscopic radiographs. *White arrows* indicate the allograft

**Fig. 13:**
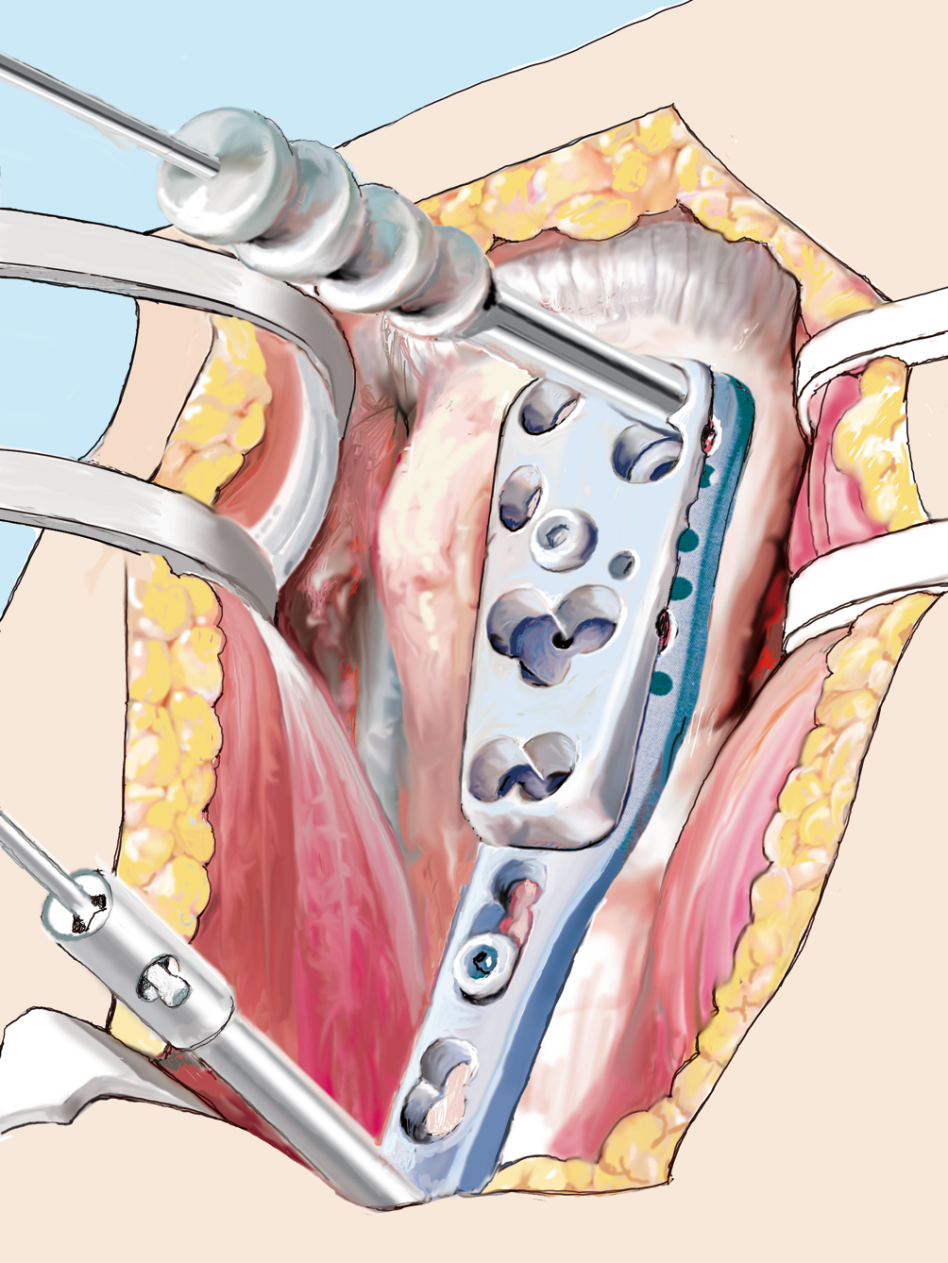
Plate attachment. Finally, an appropriately sized proximal humerus locking plate is attached directly lateral to the bicipital groove and temporarily fixed using a cortical screw in the gliding hole and two Kirschner wires. Using the fluoroscope, the plate is adjusted and positioned

**Fig. 14:**
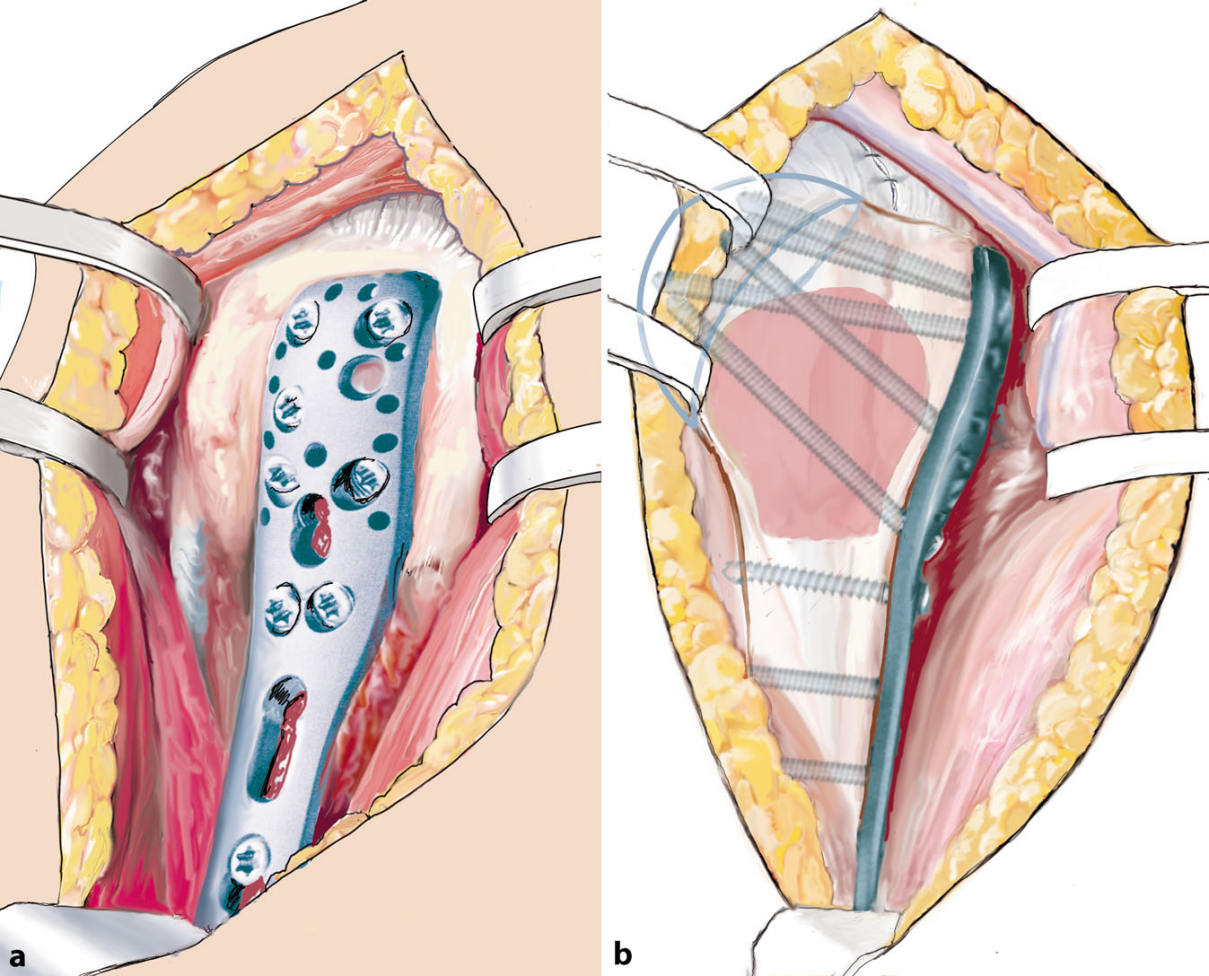
Definitive plate fixation. At least six locking head screws, including A and E levels (if at all possible to maximize the screws’ lever arm), and three bicortical shaft screws are inserted. Optionally, the functional components of the rotator cuff may be secured and knotted to the plate. Lateral (**a**) and anteroposterior (**b**) views. Allograft and humeral head highlighted translucently for better visualization

**Fig. 15:**
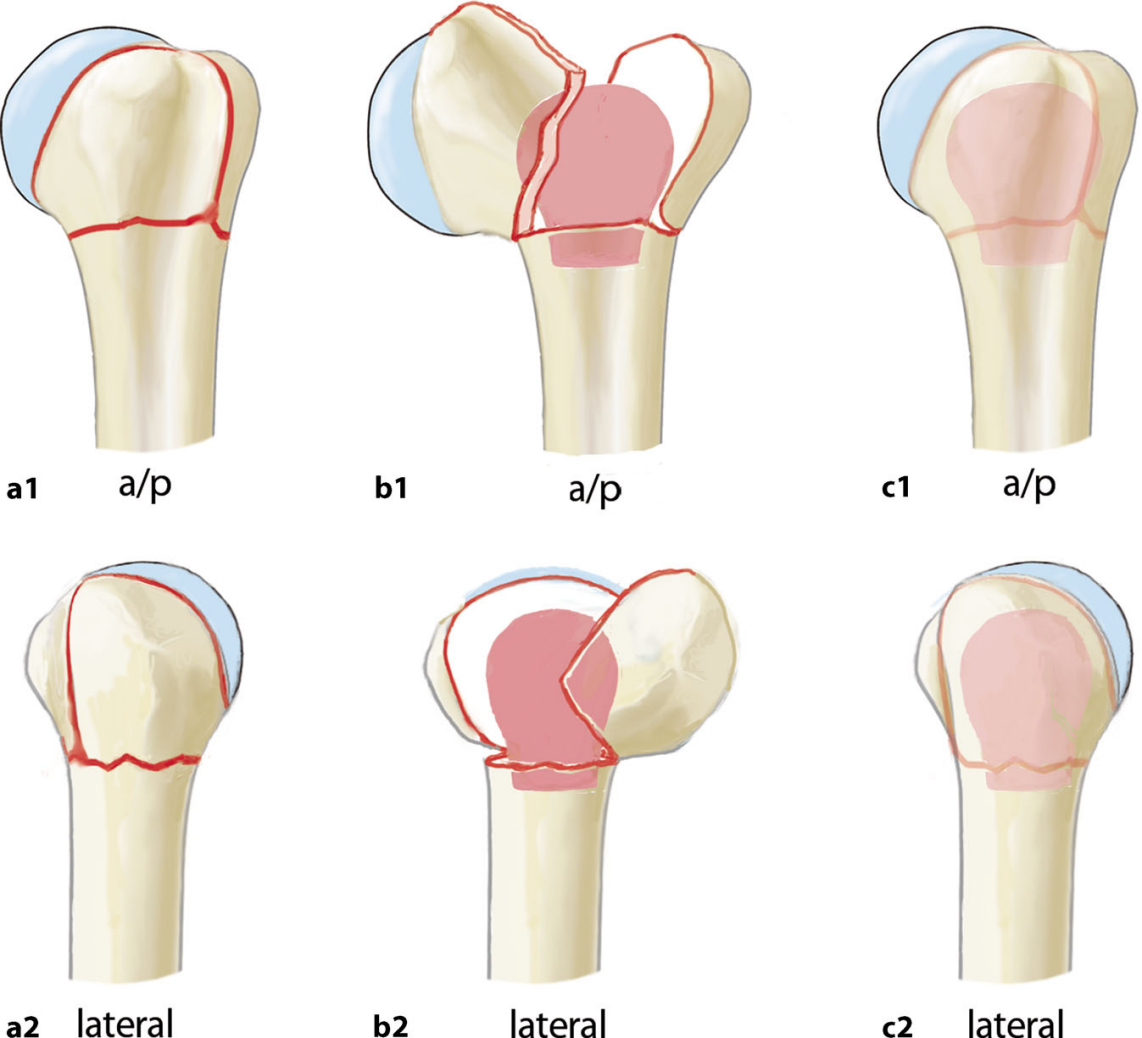
Anteroposterior (**a1, b1, c1**) and lateral (**a2, b2, c2**) schematic drawings of the preoperative (**a1,2**), intraoperative (**b1,2**), and postoperative (**c1,2**) proximal humerus. Pink “mushroom” indicating the structural allograft and its positioning within the proximal humerus in **b1,2**, and **c1,2**

**Fig. 16:**
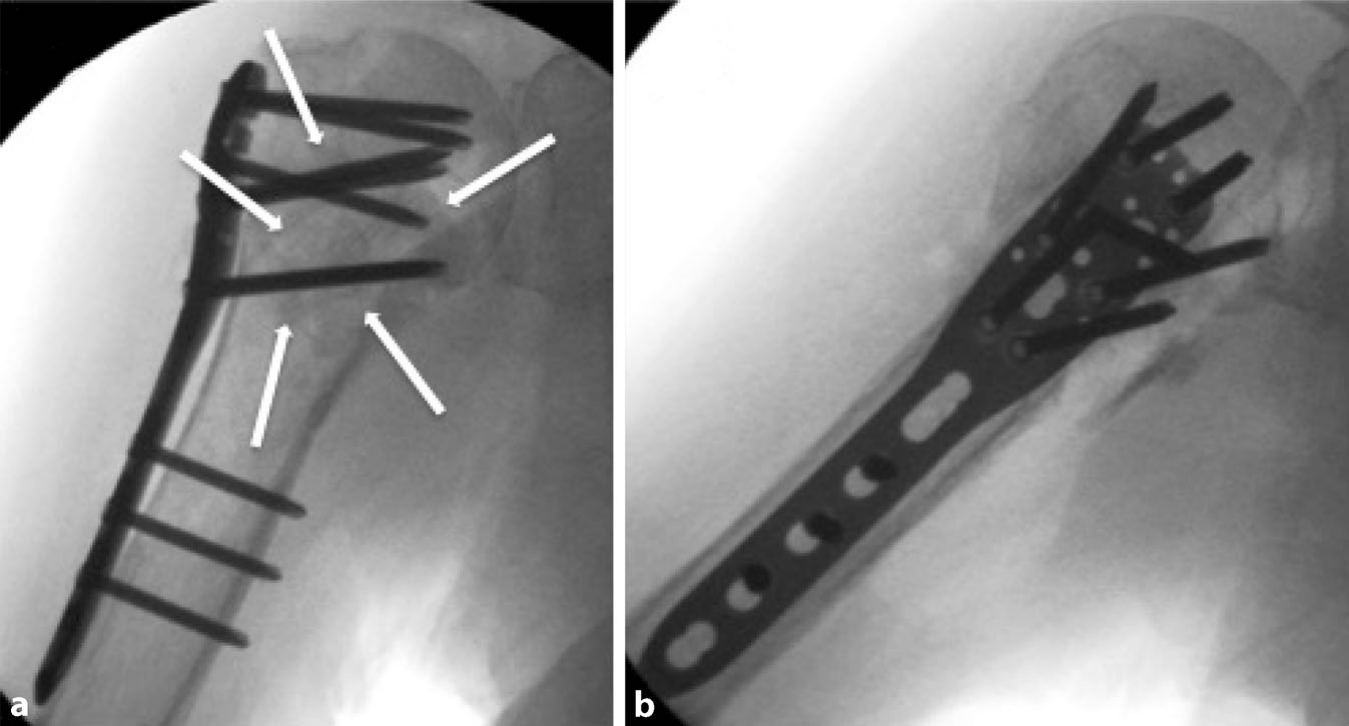
Radiological documentation. Anteroposterior (**a**) and axial (**b**) fluoroscopic views are obtained to document the proper position of the allograft and the fracture reduction. After copious lavage and hemostasis, the skin is closed in layers (subcutaneously and the skin itself, additional closure of deep structures like muscles or fascias is not recommended). Adhesive dressing, shoulder sling until interscalene block is dissolved, and for comfort afterwards (see Fig. 15 for details). Anteroposterior (**a**) and axial (**b**) fluoroscopic radiographs, intra- (post-)operatively. *White arrows* indicate allograft

**Fig. 17:**
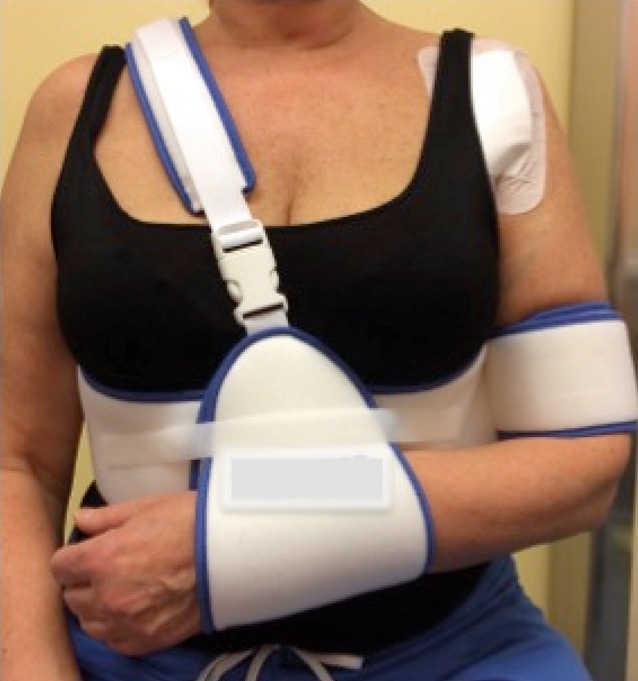
Postoperative shoulder fixation. Shoulder sling for comfort

## Postoperative management

- Removal of stitches after 12–14 days
- Cryotherapy as needed during inpatient care
- Anti-inflammatory medication on demand
- Shoulder sling for comfort
- Active assisted to active full ROM, as tolerated
- Inpatient postoperative anteroposterior (ap), outlet view (ov), and Velpeau view radiographs
- Clinical and radiological follow-ups (FU):
  - Week 6: clinical FU, ap, ov, and ax (as tolerated) radiographs
  - Months 3, 6, and 12: clinical FU, ap, ov, and ax radiographs

## Errors, hazards, complications

- Allograft cut too small: use of the cut parts as additional bony putty around the graft to achieve a press fitting construct prior to definitive fixation
- Infection of the allograft: indication for surgery and explantation of the graft; implantation of a spacer, several surgical re-looks as needed, and priming for the definitive procedure (i. e., implantation of an antibiotic loaded allograft; prosthesis)

## Results

### Methods

- Retrospective case series between July 2009 and November 2011 (Tab. 1; [1])
- Cancellous allograft was used to augment plate fixation of the fractures
- Inclusion criteria
  1. Varus displaced two-part fracture (AO A2.2; >45°, unstable eroding subsidence, impression of the shaft into the head)
  2. Interval between injury and surgery between 1 and 8 weeks following an initial trial of conservative treatment
  3. Implantation of a structural bony allograft
  4. High-risk patient
  5. Patient noncompliance

**Tab. 1:** Patients demographics and concomitant diseases

Patient no.	Gender	Age (years)	Dominant arm injured	BMI	Varus angle pre-op (°)	Follow up (months)	Risk factors	ASA score	Comments
1	F	73	No	22.5	50	36	CA, CT, OP, MA	3	Pulmonary emphysema, breast cancer
2	F	62	Yes	34.6	46	24	CA, CT, MD	4	Seizures
3	F	78	No	22.2	45	27	CA, OP, DM	4	Pancreatitis
4	F	67	Yes	20.8	51	29	CA, OP	2	
5	F	52	Yes	24.1	52	48	CT, CA, OP, PI	2	
6	M	57	Yes	38.1	45	36	DE, DM, AH	3	Plexus injury (resolved)
7	M	67	No	21.1	57	41	CA, CT, DM, AH, CL	3	Chronic liver disease (Child–Pugh B)
8	F	64	Yes	25.3	58	25	CA, OP, DM	3	
9	F	56	Yes	27.5	59	28	CT, PI	3	
10	F	62	Yes	21.2	46	24	CA, PE, DM	3	Polyarthritis
Median		63.0		23.3	50.5	28.5		3	

*ASA* American Society of Anesthesiologists; *Pre-op* preoperative; *CA* chronic alcohol abuse; *CT* chronic tobacco abuse; *OP* osteoporosis; *MA* malignoma; *MD* multiple drug abuse (psychotropics); *PI* psychotic illness; *DE* severe depression; *AH* arterial hypertension; *CL* fibrotic or cirrhotic liver disease, *BMI* body mass index

### Outcomes

- Median follow-up 28.5 months (Tab. 2)
- Nine of 10 fractures healed with incorporation of the bony allografts
- No systemic or local complications
- No significant loss of reduction or evidence of avascular necrosis of the humeral head
- Median Constant–Murley score 72.0 (range 45–86)
- Median pain on the visual analog scale 1 (range 0–7)
- Median ROM:
  - Flexion 155° (range 90–170°), abduction 168° (range 95–180°), external rotation 43° (range 30–50°)
- Flexion −13 %, abduction −14 %, external rotation −15 %, compared to the uninjured contralateral side
- Median abduction power 64 % of the uninjured side
- Median varus displacement 51° (range 45–59°) preoperatively, 4° (range −5 to 19°) intraoperatively, 13° (range 1–18°) at the time of the final follow-up
  - Improvement of 38°

**Tab. 2:** Individual results

Patient no.	Constant–Murley score	Pain (VAS)	Time to surgery (weeks)	Follow-up (months)	Flexion (°)	Bony union	Abduction (°)	External rotation (°)	Abduction power (% of uninjured side)	Varus angle intra-op (°)	Varus angle post-op (°)
1	83	0	1	36	170	Yes	120	50	79	15	16
2	48	4	2	24	90	Yes	90	45	63	2	9
3	84	0	3	27	170	Yes	120	50	89	10	18
4	64	2	3	29	140	Yes	100	35	67	5	15
5	86	0	1	48	170	Yes	120	50	53	19	21
6	84	0	6	36	160	Yes	110	40	58	0	5
7	45	7	2	41	130	No	80	30	100	−5	–
8	80	0	6	25	160	Yes	120	50	43	−3	11
9	55	5	7	28	140	Yes	80	35	39	5	7
10	58	2	8	24	150	Yes	90	40	40	3	14
Median	72.0	1	3.0	28.5	155		105	43	60.5	4	12.5

*VAS* Visual Analog Scale; *Intra-op* intraoperative; *Post-op* postoperative
